# Anti-Inflammatory Effects by Pharmacological Inhibition or Knockdown of Fatty Acid Amide Hydrolase in BV2 Microglial Cells

**DOI:** 10.3390/cells8050491

**Published:** 2019-05-22

**Authors:** Mikiei Tanaka, Kazuya Yagyu, Scott Sackett, Yumin Zhang

**Affiliations:** 1 Department of Anatomy, Physiology and Genetics, Uniformed Services University of the Health Sciences, 4301 Jones Bridge Rd, Bethesda, MD 20814, USA; mikiei.tanaka.ctr@usuhs.edu (M.T.); y3501001@edu.gifu-u.ac.jp (K.Y.); scott.sackett.ctr@usuhs.edu (S.S.); 2 Department of Chemistry and Biomolecular Science, Faculty of Engineering, Gifu University, Yanagido, Gifu 501-1193, Japan

**Keywords:** immune cells, central nervous system, *N*-acylethanolamine, siRNA, serine hydrolase

## Abstract

Fatty acid amide hydrolase (FAAH) has been recognized as a therapeutic target for several neurological diseases because its inhibition can exert neuroprotective and anti-inflammatory effects by boosting the endogenous levels of *N*-acylethanolamines. However, previous studies have shown inconsistent results by pharmacological inhibition and genetic deletion of FAAH in response to inflammation. In this study we used two inhibitors, PF3845 and URB597, together with siRNA knockdown to characterize further the effects of FAAH inhibition in BV2 microglial cells. Treatment with PF3845 suppressed lipopolysaccharide (LPS)-induced prostaglandin E_2_ (PGE_2_) production, and down-regulated cyclooxygenase-2 and microsomal PGE synthase. PF3845 reduced the expression of pro-inflammatory cytokines but had no effect on the expression of anti-inflammatory cytokines. The anti-inflammatory effects of URB597 were not as potent as those of PF3845. Knockdown of FAAH also suppressed PGE_2_ production and pro-inflammatory gene expression. Interestingly, FAAH knockdown enhanced expression of anti-inflammatory molecules in both the absence and presence of LPS treatment. The anti-inflammatory effects of FAAH inhibition and knockdown were not affected by the cannabinoid receptor antagonists or the peroxisome proliferator-activated receptor (PPAR) antagonists. Although inhibition and knockdown of FAAH have potent anti-inflammatory effects and possibly lead to the dynamic change of microglial gene regulation, the underlying mechanisms remain to be elucidated.

## 1. Introduction

The endocannabinoid (eCB) system is a complex endogenous signaling system, composed of mainly eCB ligands, their G-protein coupled receptors (CB1 and CB2), the enzymes involved in endocannabinoid biosynthesis and degradation, and the signaling pathway regulated by eCB [1]. Anandamide (AEA) and 2-arachidonylglycerol are the most widely studied eCB ligands. Numerous studies on the biological actions of these compounds indicate that the eCB system has important regulatory roles in several physiological and pathological conditions [2,3] in both the central nervous system [4] and the periphery [5]. Its physiological functions include, but are not limited to, neuroendocrine regulation [6], synaptic plasticity [7,8], bioenergetics [9], neurogenesis, and neural differentiation [4,10].

To date, it is generally accepted that eCB ligands exert their function mainly through binding to CB1 and/or CB2 receptors [11]; however, other receptors including GPR18 [12], GPR55 [13], transient receptor potential vanilloid 1 (TRPV1) [14], and peroxisome proliferator-activated receptors (PPARs) [15] are also likely to be involved in eCB signaling under physiological and pathological conditions.

Fatty acid amide hydrolase (FAAH) belongs to a large serine hydrolase family. It hydrolyzes *N*-acylethanolamines (NAEs) such as AEA, *N*-palmitoylethanolamine (PEA), and *N*-oleoylethanolamine (OEA). PEA and OEA have been shown to potentiate the activity of eCBs at their receptor targets; this activity is referred to as an entourage effect [16]. They also bind to PPARs to modulate various metabolic pathways [17]. A number of studies involving genetic or pharmacological inhibition of FAAH have demonstrated anti-nausea [18], analgesia [19], suppression of inflammatory pain [20], antidepressant effects [21], and amelioration of neurodegeneration [22] and traumatic brain injury [23,24] due to the increased levels of NAEs. Most of these studies performed in vivo demonstrate that the beneficial effects of FAAH inhibition are possibly mediated by activation of cannabinoid receptors or PPARs. From in vitro studies, several molecular mechanisms mediated by distinct receptors [25,26] and signaling pathways [27,28,29] have been reported. However, the reports on the role of FAAH inhibition on microglial inflammatory response are inconsistent between genetic deficiency and pharmacological blockade [30] and also among the different FAAH inhibitors [31]. Thus, it is necessary to characterize further the effects of FAAH inhibition and elucidate the molecular mechanisms contributing to the inflammatory response.

Microglia are the residential immune cells inside the central nervous system that work to maintain neuronal activity and immunological integrity. Under pathological conditions, microglia are activated to produce pro-inflammatory cytokines and molecules, such as eicosanoids, which recruit other immune cells including blood macrophages, and to provoke cytotoxic compounds such as reactive oxygen species (ROS), which eliminate pathogens or infected cells [32]. This activation promotes neuroinflammation and can be toxic to bystander neurons as a consequence of chronic activation. Conversely, microglia under alternative activation secrete anti-inflammatory cytokines and neuroprotective compounds to facilitate neuron proliferation and remyelination under certain pathological conditions, such as wound healing [33]. Thus, it is beneficial to regulate the transition of microglia between pro-inflammatory and anti-inflammatory phenotypes in neurological disorders. Recent reports suggest that the eCB system plays a critical role on neuroimmune regulation by modulating microglial activation [34,35]. However, it is unclear whether microglial polarization is modulated by FAAH inhibition.

In this study we investigated the anti-inflammatory response of FAAH inhibition in the BV2 microglial cell line through both pharmacological and genetic approaches. To this end, we examined two commonly-used FAAH inhibitors with distinct chemical structures, URB597 and PF3845, and transfected cells with siRNA knockdown. Our study showed that FAAH inhibition by both siRNA knockdown and FAAH inhibitors suppressed inflammatory mediators, prostaglandin E_2_ (PGE_2_), and pro-inflammatory cytokines. Expression of anti-inflammatory genes was increased by knockdown but not by inhibitors of FAAH, suggesting that different mechanisms might be attributable to the anti-inflammatory effects and microglial phenotypes induced by genetic suppression and pharmacological inhibition.

## 2. Materials and Methods

### 2.1. Reagents

The serine hydrolase activity-based protein profiling probe (ABPP probe), FP-TAMRA, was purchased from Thermo Fisher Scientific (Waltham, MA, USA). Small interfering RNAs (FlexiTube SI02736706 for fatty acid amide hydrolase (FAAH) gene knockdown and Allstars Negative Control) were purchased from Qiagen (Tokyo, Japan), and transfection reagent Lipofectamine RNAiMAX was purchased from Thermo Fisher Scientific. All inhibitors, antagonists, and reagents, including PF3845, URB597, SR141716, SR144528, GW9662, GW6471, O1918, anandamide (AEA), and arachidonic acid (AA), were purchased from Cayman Chemical (Ann Arbor, MI, USA).

Radioactive anandamide [ethanolamide-1,2-^14^C] was purchased from American Radiolabeled Chemicals, Inc (Saint Louis, MO, USA). Other chemicals including lipopolysaccharide were purchased from Sigma-Aldrich (St. Louis, MO, USA).

### 2.2. Cell Culture and Treatment

BV2 microglia cells were cultured in complete DMEM containing 10% heat inactivated fetal bovine serum (Life Technologies, Grand Island, NY, USA) under a humidified 5% CO_2_ environment. The cells were maintained by passage every other day. For cell treatment with inhibitor, cells were inoculated on culture dishes one day prior to experiment to reach 90% confluence at the beginning of treatment. Cells were incubated with antagonists for 30 min, followed by addition of FAAH inhibitors for 30 min. One hundred ng/mL of LPS were then added to the medium and incubated for 8 h for RNA analysis and 18 h for protein analysis.

### 2.3. siRNA Transfection

BV2 cells in 24-well plates (50 to 70% confluent) were transfected with siRNA for the mouse FAAH gene or negative control using Lipofectamine RNAiMAX according to the manufacturer’s protocol. Briefly, 5 pmoles of siRNA and 4.5 µL of the transfection reagent were mixed in 50 µL of OPTI-MEM for 5 min at room temperature according to the manufacturer’s protocol. The siRNA complex suspension was added dropwise to the 24-well cell culture plate. After one-day transfection, one batch of cells was incubated with LPS (100 ng/mL) for 8 h; the cells were then collected for isolation of the total RNA and preparation of the membrane fraction. A second batch of cells was incubated with LPS (100 ng/mL) for 18 h, and the cell lysates were prepared for western blotting.

### 2.4. PGE_2_ EIA

A multi-well cell culture plate was prepared one or two days prior to the test. For long-term treatment, the cell culture medium was replaced with fresh, pre-warmed medium containing the desired inhibitors and incubated for 30 min. For AEA or AA co-incubation, AEA or AA at 10 µM was added to the medium following the addition of inhibitor and then treated with 100 ng/mL of LPS. After incubation for 18 h, the cell culture medium was collected into a microtube. For short-term inhibitor treatment, cells previously activated by 100 ng/mL of LPS for 18 h were treated with the selective FAAH inhibitor and incubated for 30 min, followed by the addition of 10 µM of AEA or AA. After 30 min incubation, the cell medium was then collected into a microtube and stored at −20 °C until use. Before employing EIA, the medium was centrifuged at 5000 rpm for 2 min with a table top centrifuge to exclude residual cells and debris. Assay of PGE_2_ was performed following the manufacturer’s protocol for the PGE_2_ EIA kit (Cat# 514010, Cayman Chemical).

### 2.5. Membrane Fractionation and Activity-Based Protein Profiling (ABPP)

BV2 cells in 10 cm dishes were collected by scraping and rinsed once with chilled PBS. The cell suspension in PBS was subjected to a sonicator (model Q125 with CL-18 probe from Qsonica LLC, Newtown, CT, USA) for 15 s at 1 s intervals for every second of sonication with 45% output power. The homogenate was centrifuged at 1400× *g* for 10 min at 4 °C to remove debris and undisrupted cells, and the supernatant was transferred to a 1.5 mL Beckman ultracentrifuge tube and centrifuged at 100,000× *g* for 50 min at 4 °C. After removing the supernatant, the pellet was rinsed once with PBS and then completely suspended with 150 µL of PBS by brief sonication. The protein concentration was determined by a DC protein assay kit using bovine serum albumin as a standard according to the manufacturer’s protocol (Bio-Rad, Hercules, CA, USA) and stored at −80 °C until use. One mg/mL of the membrane fraction was pre-incubated with inhibitor at the indicated concentration for 15 min at room temperature and then mixed with FP-TAMRA (final concentration 2 µM) for 20 min at 37 °C. FP-TAMRA is a florescent probe that can covalently bind to the active, but not inactive, or the inhibitor-bound catalytic site of serine hydrolases including FAAH.

**Figure 1:**
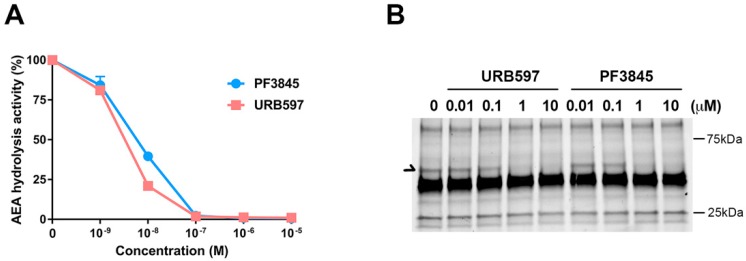
Characterization of the fatty acid amide hydrolase (FAAH) inhibitors URB597 and PF3845. AEA hydrolytic activity in BV2 membrane in the presence of inhibitor was assayed by measuring radioactive ethanolamine production by hydrolysis from [^14^C]-AEA. The plots for PF3845 dose response as a blue line and URB597 as an orange line are represented as relative activity with means ± S.D. (n = 3) (**A**). Activity-based protein profiling probe (ABPP), using the FP-TAMRA serine hydrolase probe, was performed in BV2 membrane fraction after treatment with the FAAH inhibitors. Arrow indicates the signal corresponding to FAAH (**B**).

The reaction was mixed with SDS-PAGE sampling buffer and heated at 95 °C for 5 min. Approximately 10 µg of the protein was loaded on SDS-PAGE. The gel was scanned with a fluorescence imager, ChemiDoc MP (Bio-Rad), using Cy3 mode (Epi-green light from 520 nm to 545 nm for excitation and detection of emission between 577 nm and 613 nm) to detect the active form of serine hydrolases, including FAAH, which are conjugated with FP-TAMRA in a gel (Figure 1B and Figure 6B lower panel). Subsequently, the proteins in the gel were transferred onto a nitrocellulose membrane, and western blotting was performed to assess the expression of FAAH using an anti-FAAH antibody (Figure 6B upper panel).

### 2.6. Western Blotting

For western blotting, cell lysate was prepared with RIPA buffer containing NaCl 150 mM, Tris-HCl (pH 8.0) 50 mM, 1% Triton X-100, 0.5% Na deoxycholate, 0.1% SDS, EDTA 1 mM, EGTA 1 mM, Na_3_VO_4_ 1 mM, β-glycerophosphate 1 mM, and the protease inhibitor cocktail from Roche Applied Sciences for 5 min on ice, followed by centrifugation at 12,000× *g* for 5 min at 4 °C to remove debris. The transferred nitrocellulose membrane was pre-incubated with 5% BSA in PBS+0.05% Tween 20 (PBST) for 30 min and then incubated with anti-β-actin antibody (AC-74, Sigma-Aldrich) at 1:2000, anti-iNOS antibody (Cat# 15323, Abcam, Cambridge, UK) at 1:1000, anti-FAAH antibody (Cat# 54615, Abcam) at 1:1000, or anti-COX-2 antibody (Cat# 160106, Cayman Chemical) at 1:500 in PBST at 4 °C overnight. The membrane was reacted with a secondary antibody conjugated with horse radish peroxidase (Bio-Rad) at 1:2500 for 1.5 h, followed by visualization with ECL reagent (Thermo Scientific), using an imaging system (ChemiDoc, Bio-Rad) with chemiluminescent mode. Band intensity was quantified with NIH ImageJ software.

### 2.7. qRT-PCR

Total RNA from BV2 cell cultures was isolated using TRIzol reagent according to the manufacturer’s protocol. The RNA concentration was measured by NanoDrop 1000 (Thermo Fisher Scientific), and 0.5 µg of RNA was employed for cDNA synthesis using the MAXIMA cDNA synthesis kit with dsDNase (Thermo Fisher Scientific). RNA was incubated with double strand DNase for 5 min at 37 °C in a 0.5 mL PCR tube and then mixed with reverse transcriptase. The reaction mixture was incubated in a thermal cycler (25 °C × 5 min, 50 °C × 50 min, 85 °C × 5 min). Quantitative PCR was performed in the presence of gene specific primers (250 nM of each primer) listed in Table 1 using Power SYBR Green PCR master mix (Thermo Fisher Scientific) in a 12 µL reaction mixture. In the thermal cycler, reaction mixtures were exposed to 95 °C for 10 min, followed by 40 cycles of 95 °C × 15 s and 60 °C × 60 s and then by the default melting temperature determination program installed by the LightCycler 480 system software (Roche Life Science, Indianapolis, IN, USA). The relative expression levels of the genes of interest were calculated based on the Ct value of the GAPDH gene as an internal control. Gene specific PCR amplification was confirmed by each gene’s melting curves.

### 2.8. AEA Hydrolysis Assay

Hydrolysis activity of AEA was carried out based on previous methods [36]. The membrane fraction was prepared as described above. Two hundred µL of the membrane fraction (50 µg/mL) was pre-incubated in a silanized glass tube with or without FAAH inhibitor for 30 min at 37 °C and then mixed with 20 µL of radiolabeled 0.05 µCi of ^14^C-AEA (36 µM at final concentration) in PBS containing 0.05% fatty acid-free bovine serum albumin. After 30 min incubation at 37 °C, the reaction was terminated by adding 1 mL cold Methanol/Chloroform (1:1). The mixture was briefly vortexed, then centrifuged at 3000× *g* for 5 min to separate aqueous and organic phases. The aliquot of the aqueous phase was mixed with a scintillation cocktail and measured by a scintillation counter, LS6500 (Beckman Coulter, Brea, CA, USA). The radioactivity of the sample was subtracted by that of the blank control without any membrane fraction.

### 2.9. ROS Assay

BV2 cells were plated on 96-well plates to reach 80 to 90% confluence by the next day. The medium was replaced with fresh medium containing PF3845 and 1 µM of CB1 or CB2 receptor antagonist and incubated for 30 min. LPS (100 ng/mL) was added to the medium and incubated for 8 h. The cells were washed with pre-warmed Earl’s basal salt solution (EBSS) three times and then incubated with 100 µL of pre-warmed EBSS containing 20 µM of 2’,7’-dichlorodihydrofluorescein diacetate (DCF-DA) and incubated for 30 min. The fluorescence intensity was measured by a multi-well plate reader (SpectraMax Gemini, Molecular Devices, San Jose, CA, USA) using excitation at 483 nm and emission at 535 nm. The ROS was determined by subtracting the background in the well without cells.

### 2.10. Statistics

All data are expressed as means ± SD. GraphPad Prism7 (GraphPad Software Inc., San Diego, CA, USA) was used for statistical comparisons. Statistical comparisons among multiple drug treatment groups were made using one-way ANOVA followed by Tukey’s test. In the case of siRNA knockdown cells treated with LPS or other drugs, two-way ANOVA with the Bonferroni post-test was applied. Statistical significance was set at *p* < 0.05. The symbols *, **, and *** denote *p* < 0.05, *p* < 0.01, and *p* < 0.001, respectively.

## 3. Results

### 3.1. Inhibition Profile of PF3845 and URB597 In Vitro Conditions

PF3845 and URB597 are relatively well-characterized FAAH inhibitors; however, their potency and therapeutic effects have not been examined comparatively in the same experimental settings. To determine their pharmacological properties, membrane fraction from BV2 microglial cells was incubated with the FAAH inhibitors and radiolabeled anandamide (AEA) for 30 min for measuring AEA hydrolytic activity (Figure 1A). The IC_50_ of both PF3845 and URB597 was shown to be less than 20 nM, and the hydrolysis activity was completely blocked at 1 µM. These results indicated that all AEA hydrolysis activity was attributed to FAAH in BV2 microglial cells. Similarly, using fluorophosphonate-TAMRA, an ABPP probe, to assess the serine hydrolase activity, we found that FAAH activity was inhibited in a concentration-dependent manner (Figure 1B). URB597 and PF3845 showed partial inhibition at 100 nM and complete inhibition at 1 µM. Both inhibitors did not show any cell toxicity even up to 10 µM examined by Alamar Blue cell viability and microscopic examination. There were no other serine hydrolases affected by either of these inhibitors, suggesting that both URB597 and PF3845 are highly selective FAAH inhibitors.

### 3.2. Reduction of PGE_2_ Production by FAAH Inhibitors

**Figure 2:**
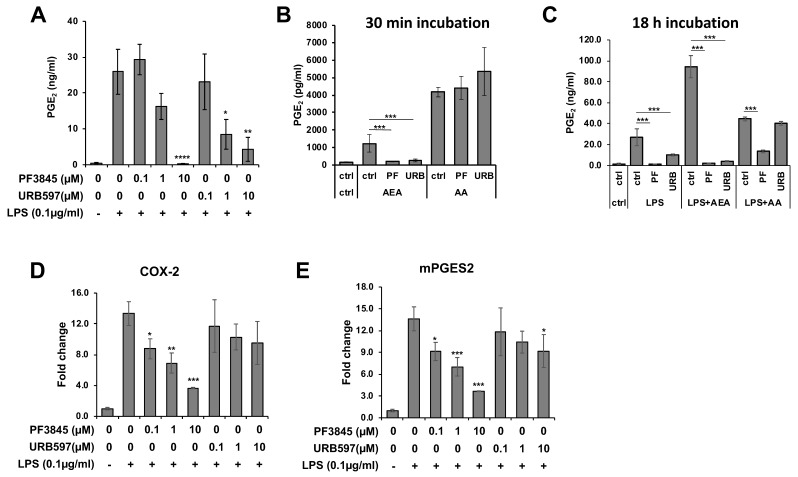
PGE_2_ production and the biosynthetic enzyme expression affected by FAAH inhibitors. BV2 cells were treated with LPS and FAAH inhibitor at the indicated concentration for 18 h, and PGE_2_ in the medium was measured by EIA (**A**). Activated BV2 cells pretreated with LPS for 18 h were incubated in the medium containing AEA (10 µM) or AA (10 µM) and PF3845 (10 µM) or URB597 (10 µM) for 30 min. PGE_2_ in the medium was assayed with EIA (**B**). BV2 cells were incubated with LPS and PF3845 (10 µM) or URB597 (10 µM) in the presence of AEA (10 µM) or AA (10 µM) for 18 h. PGE_2_ in the medium was assayed using EIA (**C**). Expression levels of COX-2 (**D**) and mPGES2 (**E**) were determined relative to GAPDH in BV2 cells after 8 h treatment with LPS and inhibitor at indicated concentrations. The + and – symbols indicate the presence or absence, respectively, of LPS. Data are presented as means ± S.D. (n = 3). *, **, and *** denote *p* < 0.05, *p* < 0.01, and *p* < 0.001, respectively.

PGE_2_ is a typical lipid mediator that is induced in response to inflammation. To examine the effects of FAAH inhibitors on inflammation, production of PGE_2_ was measured in BV2 cells treated with the FAAH inhibitors under the LPS treatment condition. Both PF3845 and URB597 dose-dependently reduced the production of PGE_2_ after 18 h incubation (Figure 2A). To determine whether the two inhibitors block the inducible PGE_2_ biosynthetic enzymes directly, the BV2 cells, which were previously incubated with LPS overnight to induce biosynthetic activity, were treated with the inhibitors for a short period of time (30 min). In the presence of AA, PGE_2_ production was not inhibited by either of these inhibitors; however, it was completely blocked when AEA was used as a substrate (Figure 2B). These results suggest that the FAAH inhibitors blocked FAAH, which converts AEA to AA, but did not directly inhibit the enzymes that metabolize AA to PGE_2_. Conversely, when cells were incubated with LPS and FAAH inhibitor in the presence of AA or AEA for a long period of time (18 h), PGE_2_ production was substantially inhibited by PF3845 not only in the presence of AEA but also in the presence of AA (Figure 2C). The reduction of PGE_2_ in the presence of AA was not affected by URB597. These results indicated that PF3845 could suppress PGE_2_ synthesis after 18 h treatment. Consistently, the mRNA expression of the chief PGE_2_ synthetic enzymes, cyclooxygenase-2 (COX-2) and microsomal PGE synthase 2 (mPGES2), was reduced dose-dependently after 18 h incubation with PF3845, whereas URB597 did not attenuate the expression of COX-2, and only at 10 µM did it show partial inhibition on the expression of mPGES2 (Figure 2D,E).

### 3.3. Reduction of Inflammatory Gene Expression by FAAH Inhibitors

**Figure 3:**
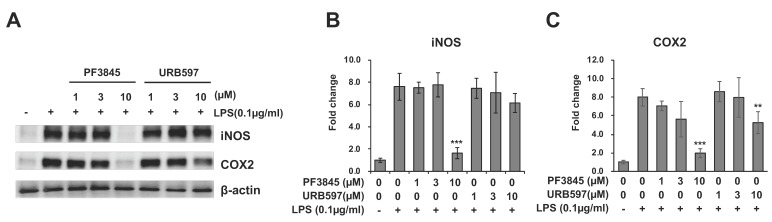
Expression of iNOS and COX-2 after FAAH inhibitor treatment. BV2 cells were treated with LPS and FAAH inhibitor for 18 h. The cell lysate was employed for western blotting with β-actin, iNOS, and COX-2 antibodies (**A**). Protein expression levels were assessed by quantifying band intensity, and relative expression to β-actin is shown in (**B**) for iNOS and in (**C**) for COX-2. The + and – symbols indicate the presence or absence, respectively, of LPS. Data are presented as means ± S.D. (n = 3). ** and *** denote *p* < 0.01 and *p* < 0.001, respectively.

To determine the effect of FAAH inhibition on inflammatory enzymes, protein expression of inducible nitric oxide synthase (iNOS) and COX-2 after incubation with LPS and inhibitor for 18 h was examined by western blotting (Figure 3A). Figure 3B,C showed the protein expression levels of iNOS and COX-2 relative to β-actin. Both enzymes were substantially reduced to nearly basal level by 10 µM of PF3845 (80% and 70% reduction in iNOS and COX-2, respectively). URB597 (10 µM) suppressed the expression of iNOS slightly and the expression of COX-2 by 50%.

**Figure 4:**
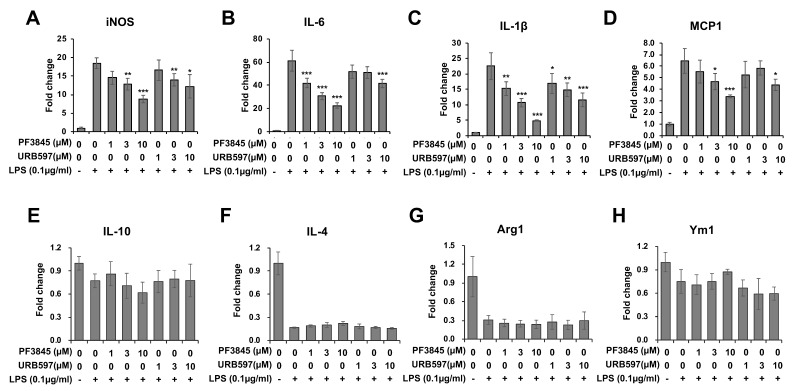
Regulation of pro-inflammatory and anti-inflammatory genes by FAAH inhibitor. BV2 cells were treated with LPS and inhibitor for 8 h. Total RNA from the cells was used for qRT-PCR to measure the expression levels of the pro-inflammatory genes, iNOS (**A**), IL-6 (**B**), IL-1β (**C**), and MCP-1 (**D**), as well as the anti-inflammatory markers, IL-10 (**E**), IL-4 (**F**), Arg1 (**G**) and Ym1 (**H**). The + and – symbols indicate the presence or absence, respectively, of LPS. Data are presented as means ± S.D. (n = 3). *, **, and *** denote *p* < 0.05, *p* < 0.01, and *p* < 0.001, respectively.

Next, we examined the mRNA expression of M1 and M2 markers including inflammatory cytokines at 8 h following LPS and inhibitor treatments (Figure 4). The expression of iNOS (Figure 4A) was suppressed by both PF3845 (up to 50% reduction) and URB597 (up to 30% reduction). All pro-inflammatory cytokines were reduced in a dose-dependent manner by PF3845 and URB597 with up to 60% and 30% reduction in IL-6 (Figure 4B), 80% and 50% reduction in IL-1β (Figure 4C), and 50% and 30% reduction in MCP1 (Figure 4D). The expression of the representative M2 markers, IL-10 (Figure 4E), IL-4 (Figure 4F), arginase-1 (Figure 4G), and Ym1 (Figure 4H) was reduced by LPS, but neither of the inhibitors blocked this reduction in LPS-treated cells.

### 3.4. Anti-Inflammatory Effects of FAAH Inhibitors Independent of CB Receptor Activation

**Figure 5:**
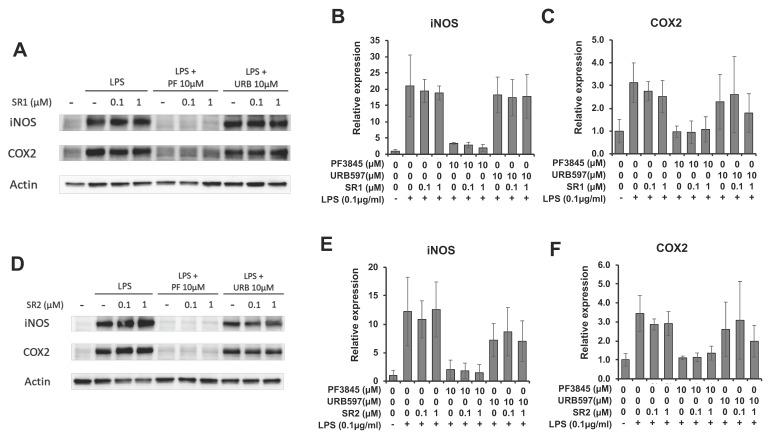
Effects of CB receptor antagonists on the FAAH inhibitor-mediated anti-inflammatory response. BV2 cells were treated with LPS, FAAH inhibitor (10 µM), and the CB1 antagonist, SR1 (1 µM), for 8 h. The cell lysate was used for western blotting with β-actin, iNOS, and COX-2 antibodies (**A**). The relative expression of iNOS (**B**) and COX-2 (**C**) is shown after normalizing with β-actin expression. BV2 cells were treated with LPS, FAAH inhibitor, and the CB2 antagonist, SR2 (1 µM), for 8 h, and western blotting was performed for iNOS and COX-2 together with β-actin (**D**). Relative expression levels are shown for iNOS in (**E**) and COX-2 in (**F**). The + and – symbols indicate the presence or absence, respectively, of LPS. Data are presented as means ± S.D. (n = 3).

The anti-inflammatory mechanism by FAAH inhibitors was characterized by treatment with antagonists for CB receptors. Co-incubation with the CB1 receptor antagonist, SR141716 (SR1), did not reverse the reduction of COX-2 or iNOS expression caused by the FAAH inhibitors (Figure 5A–C). The CB2 receptor antagonist, SR144528 (SR2), also did not affect the expression of COX-2 or iNOS (Figure 5D–F). In the presence of URB597, neither CB1 nor CB2 receptor antagonist showed any clear effects due to large sample variations and partial inhibition by URB597. Effect of the antagonists was also assessed based on the generation of reactive oxygen species (ROS) using DCF-DA (Supplementary Figure S1). ROS production was dose-dependently suppressed by PF3845 and reduced to near basal level at 1 µM PF3845. However, the suppression of ROS was not reversed by the CB receptor antagonists. ROS production was further reduced by co-treatment with SR1, suggesting that SR1 *per se* may also have anti-inflammatory effects as we previously reported [37]. Thus, the CB receptors were not involved in the anti-inflammatory response of FAAH inhibition based on ROS production and protein expression of the inflammatory enzymes.

### 3.5. Construction of FAAH Knockdown Cells

**Figure 6:**
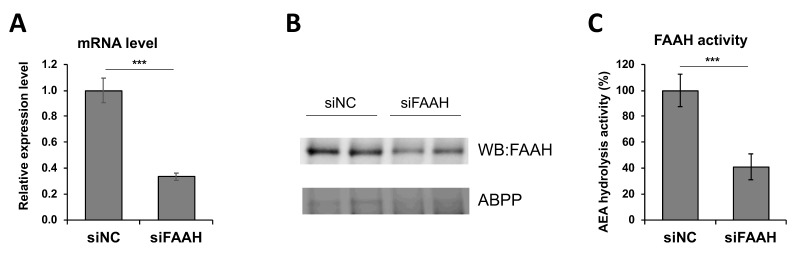
Characterization of FAAH siRNA knockdown cells. BV2 cells were transfected with siRNA for FAAH or negative control followed by 24 h incubation. FAAH mRNA expression was determined by qRT-PCR relative to GAPDH (**A**), and FAAH protein expression was assessed by western blotting and ABPP with FP-TAMRA probe (**B**). AEA hydrolysis activity in the membrane was measured as shown in Figure 1A (**C**). Data are presented as means ± S.D. (n = 3). *** denotes *p* < 0.001.

To investigate the effect of genetically-modified expression of FAAH, knockdown cells were constructed by siRNA transfection. As shown in Figure 6A, the siRNA transfected cells showed a 60% reduction in the FAAH transcript compared with negative control siRNA transfected cells. Protein expression of FAAH was decreased accordingly based on western blotting and ABPP assay (Figure 6B). AEA hydrolysis activity was also assessed using the membrane fraction from the cells after 24 h siRNA transfection. Consistent with suppressed expression of FAAH mRNA and protein (Figure 6A,B), the FAAH siRNA transfected cell membrane showed a 60% reduction in the AEA hydrolytic activity compared with the negative control siRNA cell membrane (Figure 6C). Collectively, the FAAH knockdown cells exhibited approximately a 60% reduction in FAAH expression and its enzymatic activity.

### 3.6. Reduced Inflammatory Responses but Increased M2 Markers by FAAH Knockdown.

**Figure 7:**
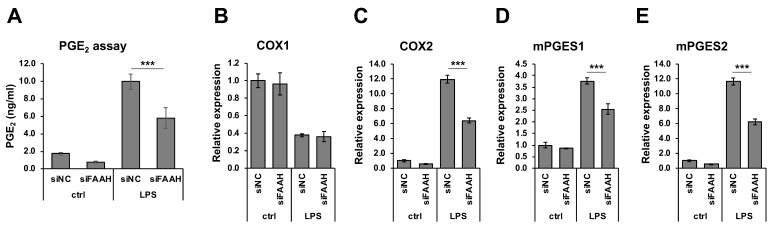
PGE_2_ production and biosynthetic enzyme expression in FAAH knockdown cells. BV2 cells after 24 h siRNA transfection were incubated with LPS for 18 h. The medium was employed for PGE_2_ EIA (**A**). For mRNA expression analysis, BV2 cells after 24 h siRNA transfection were treated with LPS for 8 h. qRT-PCR was performed to determine expression of the PGE_2_ biosynthetic enzymes COX-1 (**B**), COX-2 (**C**), mPGES1 (**D**) and mPGES2 (**E**). Graphs are represented as means ± S.D. (n = 4) determined by two-way ANOVA. *** denotes *p* < 0.001.

**Figure 8:**
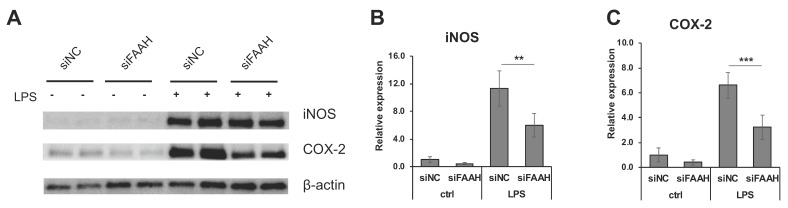
Expression of iNOS and COX-2 in FAAH knockdown cells. BV2 cells after 24 h siRNA transfection were treated with LPS for 18 h, and the cell lysates were collected. Western blotting was performed for iNOS, COX-2, and β-actin (**A**). Relative expression levels to β-actin are shown for iNOS in (**B**) and for COX-2 in (**C**). The + and – symbols indicate the presence or absence, respectively, of LPS. Data are presented as means ± S.D. (n = 5) determined by two-way ANOVA. **, and *** denote *p* < 0.01 and *p* < 0.001, respectively.

FAAH knockdown cells showed reduction of PGE_2_ production compared with the negative control cells both with and without 18 h LPS treatment (Figure 7A). Consistent with the reduced PGE_2_ production, mRNA expression of the PGE_2_ biosynthetic enzymes (COX-2, mPGES1, and mPGES2) was significantly down-regulated in the knockdown cells after LPS treatment (Figure 7C–E). The expression of COX-2 and mPGES2 was also decreased in the absence of LPS treatment. On the other hand, the expression of COX-1 was not altered by FAAH knockdown (Figure 7B). Consistently, the protein expression of iNOS in the LPS-treated cells was significantly reduced in FAAH knockdown cells (Figure 8A,B). Notably, COX-2 expression was decreased significantly in knockdown cells both with and without LPS treatment (Figure 8A,C).

**Figure 9:**
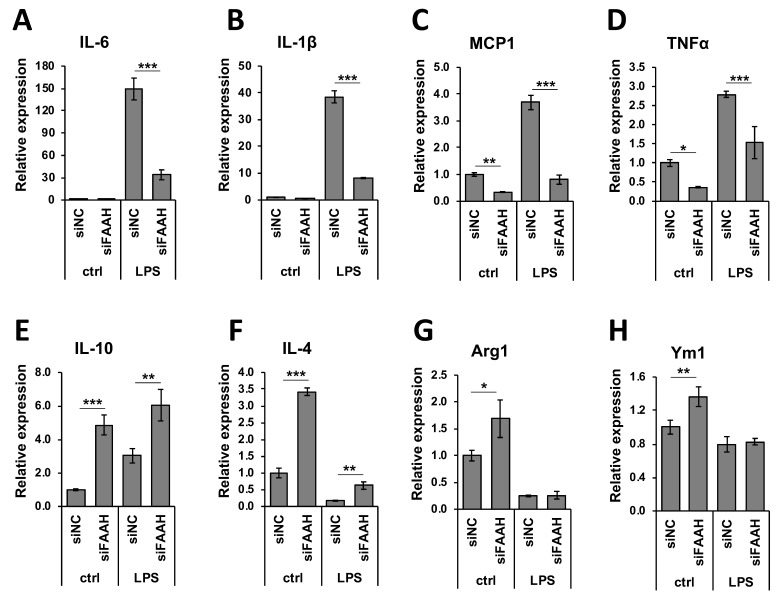
Expression of pro- and anti-inflammatory markers in FAAH knockdown cells. Transfected cells with siRNA were treated with LPS for 8 h and total RNA was collected. Total RNA was used for qRT-PCR to assess expression of the pro-inflammatory cytokines, IL-6 (**A**), IL-1β (**B**), MCP1 (**C**), and TNFα (**D**), as well as the anti-inflammatory markers, IL-10 (**E**), IL-4 (**F**), Arg1 (**G**), and Ym1 (**H**). Data are presented as means ± S.D. (n = 3) determined by two-way ANOVA. *, **, and *** denote *p* < 0.05, *p* < 0.01, and *p* < 0.001, respectively.

We further examined the anti-inflammatory gene regulation in FAAH knockdown cells by qRT-PCR. Treatment with LPS dramatically increased the expression of pro-inflammatory cytokines (Figure 9A–D), and FAAH knockdown reduced the expression of IL-6, IL-1β, and MCP1 by approximately 80% and TNF-α by 50%. The expression of MCP1 and TNF-α was also down-regulated by FAAH knockdown to 20% and 30%, respectively, of the total level without LPS treatment (Figure 9C,D). In the case of anti-inflammatory cytokines or M2 markers, the knockdown cells up-regulated all of these genes compared to the control siRNA transfected cells. Unlike the increased expression of pro-inflammatory cytokines, the expression of the anti-inflammatory cytokines IL-10, IL-4, Arg1, and Ym1 was dramatically reduced in LPS-treated cells in both control and siRNA transfected cells. However, FAAH siRNA knockdown increased the expression of IL-10 (2.0-fold) and IL-4 (3.6-fold) when compared to the negative control siRNA transfected cells following LPS treatment, but the expression of Arg1 and Ym1 remained unchanged (Figure 8E–H). These results indicate that knockdown of FAAH suppresses pro-inflammatory gene expression but increases the expression of anti-inflammatory genes and M2 markers.

### 3.7. Anti-Inflammatory Effects of FAAH Inhibition and Knockdown Independent of CB Receptors and PPARs

To examine whether the anti-inflammatory effects of FAAH inhibition and knockdown are mediated by the activation of CB receptors or PPARs, PF3845-treated and FAAH knockdown cells were incubated with LPS and the respective antagonists for 8 h. Table 2 and Table 3 showed relative mRNA expression levels of three inflammatory genes: COX-2, IL-1β, and MCP1. As shown previously, the expression of these genes was significantly reduced by PF3845 treatment and FAAH knockdown. The CB1 and CB2 receptor antagonists, SR1 and SR2, did not reverse the suppressive effect of either PF3845 or the FAAH knockdown.

The antagonists for PPARα, PPARγ, and the non-canonical CB receptors (GPR18 and GPR55), GW6471, GW9662, and O1918, also had no effect on the expression of these inflammatory cytokines in FAAH inhibitor-treated or the FAAH siRNA knockdown cells.

## 4. Discussion

This study demonstrates that pharmacological inhibition of FAAH suppresses the expression of several pro-inflammatory genes induced by LPS, the inducer of representative inflammatory response in vitro, which is mainly mediated by TLR4 activation in BV2 cells. PGE_2_ is a lipid mediator that activates immune cells as well as neuronal cells into an inflammatory state. We found that both PF3845 and URB597 reduced PGE_2_ production after 18 h LPS treatment, whereas these inhibitors did not directly block the activity of PGE_2_ biosynthetic enzymes when tested for short-term treatment, suggesting that the reduction of PGE_2_ was mainly due to the suppressed expression rather than the blockage of the PGE_2_ biosynthetic enzymes. To support this hypothesis, the expression of COX-2 and mPGES1 and 2 was down-regulated after overnight treatment. In addition to PGE_2_ production and the expression of several pro-inflammatory cytokines such as IL-1β, MCP1, and IL-6 was significantly reduced by the inhibitors. Notably, suppression of those inflammatory genes by PF3845 was more efficient than by URB597; e.g., the iNOS protein expression was substantially suppressed by PF3845, but only minimally by URB597. In line with the relatively inefficient efficacy of URB597, treatment with less than 10 µM of URB597 showed no effects on PGE_2_ production [38] or NO production [39] in primary microglia although both inhibitors block the catalytic site of FAAH irreversibly with comparable IC_50_ in the BV2 cell membrane (Figure 1). Although both inhibitors are permeable to the blood-brain barrier, they are structurally distinct inhibitors: URB597 is a carbamate [40,41], while PF3845 is a piperidine urea inhibitor [42,43]. We also found that PF04457845, another piperidine urea inhibitor [44], had similar anti-inflammatory effects compared to PF3845 (unpublished data). Thus, it is possible that the anti-inflammatory effects are impacted by their chemical structures or inhibitor-specific properties.

In line with this premise, a recent study showed that reduction of tyrosine hydrolase gene expression by URB597 was not replicated by other FAAH inhibitors [31], suggesting that URB597 has an alternative pharmacological effect independent of its inhibition of FAAH, even though URB597 is reported to have no off-target interaction with any receptors, transporters, ion channels, or enzymes at 10 µM concentration in another study [40]. In regard to PF3845, we did not observe any interaction with other serine hydrolases except for the expected target, FAAH, based on the ABPP analysis (Figure 1B); however, it is possible that PF3845 can interact with non-serine hydrolase enzymes. Our results, together with previous findings, imply that the FAAH-independent mechanisms might also contribute to the anti-inflammatory effects of these inhibitors.

In order to determine further the effects of FAAH modulation in inflammatory response, FAAH gene expression was knocked down by siRNA to 60% compared to the negative control siRNA. Accordingly, protein expression and AEA hydrolysis activity were also significantly reduced. Cells with FAAH knockdown showed robust anti-inflammatory effects not only under normal conditions but also following LPS activation. It is notable that transient suppression of FAAH leads to substantial suppression of pro-inflammatory genes even though 40% of the enzymatic activity still remained. Furthermore, FAAH knockdown increased the expression of several representative anti-inflammatory markers including IL-4 and IL-10 under either LPS-treated or normal conditions. Therefore, FAAH knockdown does not only suppress pro-inflammatory response, but it also shifts microglia to anti-inflammatory phenotypes. Consistent with previous reports that treatment with AEA facilitates anti-inflammatory gene expression but suppress pro-inflammatory cytokines in primary microglia [34,45], our results suggest that AEA is a critical signaling molecule to regulate microglial activation state. Moreover, recent studies found that CB2 receptor activation attenuated microglial activation in vitro through PKC activation [46] and in vivo disease models mediated by the cAMP/PKA pathway [47]. Our previous report also showed that an M2 marker, arginase-1, was up-regulated after treatment with PF3845 along with an increase in the levels of AEA in the traumatic brain injury (TBI) mouse model. Thus, modulation of the eCB system can modulate microglia toward an anti-inflammatory phenotype. It might be critical to further investigate microglia polarization induced by FAAH knockdown to identify the eCB signaling pathway and to determine the microglia phenotype not only based on gene regulation but also based on functional alteration, such as changes in phagocytosis, antigen presentation, or metabolic balance between glycolysis and respiratory chain.

Since FAAH inhibition can increase the AEA, PEA, and other congeners it is reasonable to speculate that the receptors for these lipids are involved in the anti-inflammatory effects. However, the cannabinoid receptor and PPAR antagonists had no effect on the anti-inflammatory response induced by either inhibitor or siRNA knockdown, suggesting that the FAAH inhibitory effect was independent of CB receptors and PPARs. It has been suggested that augmentation of NAEs by FAAH inhibition may interact with several ion channels expressed in BV2 microglia cells, such as the potassium channel, Kv1.5 [48], or the L-type calcium channel [26], which modulates microglial pro-inflammatory activity [49]. Besides NAEs, FAAH also catalyzes the degradation of N-acyl taurines [50]. In fact, the lipid species are found abundantly in the brain, comparable to the amount of PEA or OEA, and they are increased in FAAH knockout mice and after treatment with PF3845. Saturated N-acyl taurines can modulate the TRP family channels, TRPV1, and TRPV4, to mobilize intracellular calcium in vitro [51], or to facilitate skin wound healing [52]. Recently, Dr. Muccioli’s group reported that ω-3 NAEs such as N-docosahexaenoylethanolamine or N-docosatetraenoylethanolamine were accumulated in macrophages after treatment with PF3845 [53]. Of note, the anti-inflammatory effects of those NAEs on cytokine and iNOS expression were independent of the activation of CB1/CB2 receptors, PPARα, and PPARγ, which is reminiscent of our findings. Thus, other types of lipid molecules and their receptors could contribute to the anti-inflammatory response by FAAH inhibition.

Because of the inconsistencies in previous studies that used pharmacological inhibitors or targeted gene deletion of FAAH [30,31], in this study we characterized the role of FAAH inhibition on the anti-inflammatory response by using two structurally distinct inhibitors and genetic suppression. In previous studies, astrocytes isolated from FAAH knockout mice but not URB597-treated astrocytes were found to be more responsive to Aβ-induced pro-inflammatory cytokine expression [30]. Their subsequent study revealed that the exaggerated inflammatory response of FAAH depletion was mediated by IL-1β specifically in the transgenic Alzheimer’s disease model but not in wild type animals [54]. Thus, it is anticipated that the role of FAAH may vary depending on the neuropathological conditions. It has been suggested that TBI is a risk factor for the late onset of Alzheimer’s disease after several years of incident. Our recent study indicated that FAAH inhibition not only ameliorates microglial inflammation and pathological features including phosphorylated Tau protein in the TBI model one week post injury but also improves spatial learning and memory and synaptic integrity one month post injury [23], suggesting a possibility that Alzheimer’s disease induced by TBI can be prevented with FAAH inhibition. In this study, we found that M2 marker expression was increased by FAAH knockdown but not by pharmacological inhibition. These results suggest that different regulatory mechanisms are likely involved in these conditions. Although the molecular mechanisms activated by FAAH inhibition are still elusive, our study suggests that FAAH is a promising therapeutic target for various neurological disorders in which neuroinflammation plays a pivotal role.

## Figures and Tables

**Table 1:** Primer sequences for qRT-PCR.

Gene ^1^	Sense Primer	Anti-Sense Primer
*Arg1*	AGCCAATGAAGAGCTGGCTGGT	AACTGCCAGACTGTGGTCTCCA
*Ptgs2(Cox2)*	GAAATGGCTGCAGAATTGAA	AAGGAGAATGGTGCTCCAAG
*Faah*	CAACTACACCATGCCCACTC	GACCTCCAGGGCATAAGGTA
*Gapdh*	AGGTCGGTGTGAACGGATTTG	TGTAGACCATGTAGTTGAGGTCA
*Il4*	GGTCTCAACCCCCAGCTAGT	GCCGATGATCTCTCTCAAGTGAT
*Il6*	TAGTCCTTCCTACCCCAATTTCC	TTGGTCCTTAGCCACTCCTTC
*Il10*	GCTCTTACTGACTGGCATGAG	CGCAGCTCTAGGAGCATGTG
*Il1b*	GCAACTGTTCCTGAACTCAACT	ATCTTTTGGGGTCCGTCAACT
*Nos2 (iNOS)*	ACCTTGTTCAGCTACGCCTT	CATTCCCAAATGTGCTTGTC
*Ccl2 (MCP1)*	TTAAAAACCTGGATCGGAACCAA	GCATTAGCTTCAGATTTACGGGT
*Ptges (mPGES1)*	TGTCCAAATCCTGTCTTCCA	GGTTCTGGAGCACACCCTAT
*Ptges2 (mPGES2)*	GAAATGGCTGCAGAATTGAA	AAGGAGAATGGTGCTCCAAG
*Tnf (TNF-α)*	CCCTCACACTCAGATCATCTTCT	GCTACGACGTGGGCTACAG
*Ym1*	CAGGTCTGGCAATTCTTCTGAA	GTCTTGCTCATGTGTGTAAGTGA

^1^ For several genes listed in the table, the alternative gene names are also given in parenthesis.

**Table 2:** Effect of CB receptor or PPAR antagonist to anti-inflammatory response by PF3845 in LPS.

Treatment	COX-2	IL-1β	MCP1
LPS	1.00 ± 0.05	1.00 ± 0.07	1.00 ± 0.06
LPS+PF	0.34 ± 0.01 ^1^	0.16 ± 0.00 ^1^	0.30 ± 0.00 ^1^
LPS+PF+SR1	0.29 ± 0.02	0.16 ± 0.00	0.26 ± 0.01
LPS+PF+SR2	0.35 ± 0.02	0.16 ± 0.01	0.26 ± 0.02
LPS+PF+GW6471	0.21 ± 0.04	0.06 ± 0.01	0.24 ± 0.04
LPS+PF+GW9662	0.39 ± 0.03	0.19 ± 0.01	0.32 ± 0.04
LPS+PF+O1918	0.39 ± 0.01	0.19 ± 0.01	0.33 ± 0.03

^1^ All genes expression was significantly reduced by PF3845 (*p* < 0.01).

**Table 3:** Effect of CB receptor or PPAR antagonist in LPS-activated FAAH knockdown cells.

Treatment	COX-2	IL-1β	MCP1
siNC	1.00 ± 0.22	1.00 ± 0.25	1.00 ± 0.18
siFAAH	0.44 ± 0.12 ^1^	0.25 ± 0.11 ^1^	0.28 ± 0.10 ^1^
siFAAH+SR1	0.37 ± 0.11	0.15 ± 0.06	0.32 ± 0.04
siFAAH+SR2	0.33 ± 0.04	0.15 ± 0.05	0.26 ± 0.05
siFAAH+GW6471	0.30 ± 0.05	0.13 ± 0.04	0.32 ± 0.04
siFAAH+GW9662	0.38 ± 0.09	0.16 ± 0.05	0.34 ± 0.08
siFAAH+O1918	0.42 ± 0.12	0.20 ± 0.07	0.37 ± 0.05

^1^ All genes expression was significantly reduced by knockdown (*p* < 0.01).

## Supplementary Materials

The following are available online at https://www.mdpi.com/2073-4409/8/5/491/s1, Figure S1: Effects of PF3845 and the CB receptor antagonists on ROS production.
