# Supplementary material for: Anti-Inflammatory Effects by Pharmacological Inhibition or Knockdown of Fatty Acid Amide Hydrolase in BV2 Microglial Cells
Source: Cells. 2019 May 22;8(5):491. doi: 10.3390/cells8050491 (PMC6562696; doi:10.3390/cells8050491)
Supplement: Supplementary file 1 [file cells-08-00491-s001.pdf]

cells

MDPI

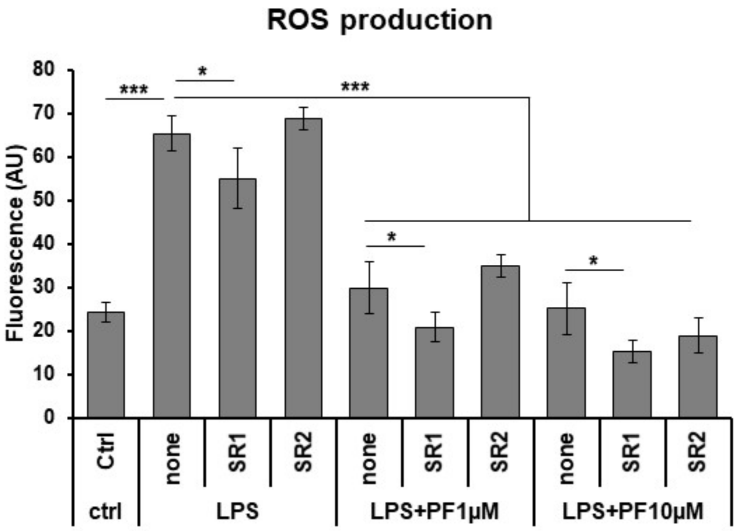

Figure S1. Effects of PF3845 and the CB receptor antagonists on ROS production. BV2 cells were treated with LPS, PF3845 (1 or 10 μM), and SR1 (1 μM) or SR2 (1 μM) for 8 h. Cells were incubated with DCF-DA for 30 min. ROS derived from LPS activated cells was determined by subtracting the background in the well without cells. PF3845 significantly decreased ROS production both with and without CB antagonists. ROS production was reduced by addition of SR1 independent on the effect of PF3845. Results are presented as means ± S.D. (n=4 to 5). *, **, and *** denote $p < 0.05$, $p < 0.01$, and $p < 0.001$, respectively.
